# Embrapa Network for Brazilian Plant Genetic Resources Conservation

**DOI:** 10.1089/bio.2018.0044

**Published:** 2018-10-12

**Authors:** Alfredo Augusto Cunha Alves, Vânia Cristina Rennó Azevedo

**Affiliations:** ^1^ Embrapa Cassava & Fruits (CNPMF), Cruz das Almas, Brazil.; ^2^ Embrapa Genetic Resources & Biotechnology (CENARGEN), Brasília-DF, Brazil.

**Keywords:** active genebank, seed collection, germplasm exchange, *ex situ* conservation, genebank database

## Abstract

Brazil is one of the most biodiverse countries on Earth, holding ∼10% of the world's vascular plant species. Despite that, Brazilian agriculture is highly dependent on genetic resources originating from other countries. Embrapa (Brazilian Agricultural Research Corporation) is the governmental institution that, since 1973, has been responsible for the introduction and conservation of genetic resources in Brazil. In this article, we report on the experiences that Embrapa has faced over the past 45 years to build and improve a national network for the preservation of plant genetic resources under the coordination of *Embrapa Genetic Resources & Biotechnology* (CENARGEN), one of the 42 Embrapa decentralized units. The first network-based model, RENARGEN, initiated in 2003, was followed by the National Platform for Genetic Resources (Platform RG) in 2009; and from 2014 until today Embrapa manages the conservation of genetic resources through Portfolio REGEN, in which the plant component is called Plant Genetic Resources Network (RGV). This network covers activities of enrichment, conservation, characterization, and documentation of genebanks. Embrapa's plant genetic resources are conserved in active genebanks (AGs), in long-term seed bank (Colbase), and *in vitro* and DNA banks. *In situ* and *on-farm* conservation are also handled at Embrapa to complement and reinforce *ex situ* conservation. The latest survey reveals that Embrapa has 134 AGs with ∼150,000 accessions of 1130 plant species, 123,000 accessions of 735 species within Colbase, 1250 *in vitro* accessions, and 12,000 DNA samples. At least 65% of this collection is documented and available to the public in the Embrapa Alelo system, which also handles quarantine, germplasm exchange, and herbarium data. By the end of 2018, the public Alelo data will be automatically migrated to the Genesys system. In the last 40 years, ∼650,000 accessions have been exchanged by Embrapa, with 70% of them imported from other countries.

## Introduction

Brazil is one of the most biodiverse countries on Earth. Its advantageous location (between 5°N and 33°S and between 35°E and 74°W) and its large continental dimension (it is the largest country in the southern hemisphere and the fifth largest in the world in total area) may be the geographic attributes that provide Brazil with the following characteristics:

- Wide climate diversity, with tropical (predominant) and subtropical regions, and derivations of these climates;
- Six vegetation biomes: Amazônia (Amazon Rainforest), Cerrado (Central Savanna), Caatinga (Thorny Forest), Atlantic Forest, Pampas, and Pantanal (Wetlands)^1^;
- Brazil has 33,161 species of vascular plants, which represents ∼25% of the Americas and 10% of the world plant diversity^2^;
- Brazil is the main center of origin of the new species of vascular plants identified in the last three decades^3^;
- 30% of Brazilian territory is arable land.
- The largest fresh water reserves on the planet (8% of the world volume).

Several Brazilian native species are used as human food, of which we highlight cassava (*Manihot esculenta*), pineapple (*Ananas comosus*), peanuts (*Arachis hypogaea*), cocoa (*Theobroma cacao*), cashew (*Anacardium occidentale*), cupuassu (*Theobroma grandiflorum*), passion fruit (*Passiflora edulis*), Brazil nuts (*Bertholletia excelsa*), guaraná (*Paullinia cupana*), jaboticaba (*Plinia cauliflora*), and some palms such as assai (*Euterpe oleracea*), which is today widely consumed all over the country and exported.^4^ In addition, native forage species are predominantly used in the raising of livestock. On the contrary, Brazilian agriculture is highly dependent on genetic resources originating from other countries. The systematic introduction of exotic genetic resources, the ecological diversity, and the development of technologies and new varieties adapted to the different Brazilian biomes resulted in an intense and ample adaptation and expansion of several crops that are today considered, globally, as basic commodities. Today, Brazil stands out as the third largest producer of grains and the third largest exporter of agricultural products in the world, ranking first in the export of coffee, soybean, sugar, orange juice, alcohol, tobacco, beef, and chicken meat.^5^

The Brazilian Agricultural Research System (SNPA), coordinated by Embrapa (Brazilian Agricultural Research Corporation), is made up of public, federal, and state institutions; universities; private companies and foundations that conduct research in different scientific areas and different geographic regions. Embrapa is linked to the Ministry of Agriculture and Food Supply, and has 42 decentralized units (DUs) distributed throughout the country. Since its founding in 1973, Embrapa was designated to promote and facilitate the safe introduction of genetic resources. *Embrapa Genetic Resources & Biotechnology* (CENARGEN) was created in 1974 to coordinate the activities of collection, conservation, quarantine, exchange, characterization, evaluation, and documentation of genetic resources in a collaborative way with SNPA partner institutions.^4^ Since then, collections of germplasm have been structured in different DUs, predominantly for plants.

Brazil has achieved important results in agricultural research, with significant progress in the conservation and use of plant genetic resources, especially those related to food production. In this article, we report on the experiences that Embrapa has faced in the construction of a national network for the conservation and use of Brazilian plant genetic resources.

## Maintenance and Conservation of Brazilian Plant Genetic Resources

### Background

The preservation of genetic resources is essential for maintaining the natural genetic variability for use in breeding programs, especially those for food. In Brazil, this need is especially important as the predominant country's food-based crops are of foreign origin. We can mention, for example, that 90% of all species conserved in Embrapa's plant collections are of exotic species.^6^

Starting in 2003, Embrapa has operated the first network-based model of management and conservation of genetic resources in a network called National Genetic Resources Network (RENARGEN). Considering the relevance of genetic resources as strategic assets for agricultural development, this model was improved and, in 2009, RENARGEN was replaced by the National Platform for Genetic Resources formed by four big project networks: (1) plant network, (2) animal network, (3) microbial network, and (4) network integration (transverse network).^7^ The first three network projects focused on the conservation of genetic resources, while the fourth project network was composed of three crosscutting project components: curatorship, documentation, and germplasm exchange, which had a strong interaction with the other component projects of the three networks.^7^

From 2012 to 2015, Embrapa established new corporate management instruments called “Portfolios” to organize projects related to strategic themes of national relevance defined directly by the Board of Embrapa.^8^ Of the 20 corporate portfolios approved during this period, the Strategic Management of Genetic Resources for Food, Agriculture and Bio-Industry (REGEN portfolio) was established in 2014.^8^ In this new model, the plant component, now called Plant Genetic Resources Network (RGV), is implementing a new phase following a restructuring of component projects. The activities of collecting wild relatives and native species of importance for agriculture and food are being reinforced, as well as *in situ*/*on-farm* conservation actions in support of *ex situ* conservation.^9^ The organization and documentation of information in the collections is a priority, and the main innovation in the RGV is the implementation of Quality Systems based on international standards, which is being established from the selection of minimum requirements.^9^

**FIG. 1.**
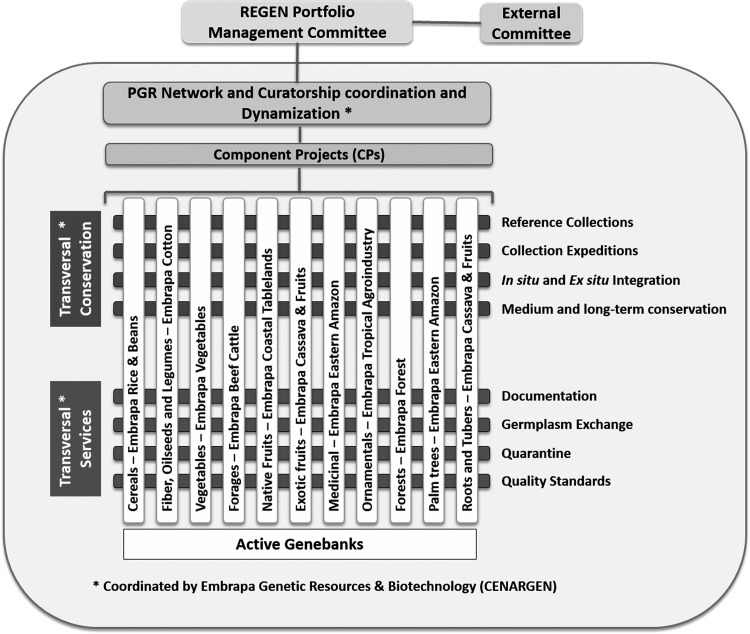
Scheme of the structure and management of the Embrapa Plant Genetic Resources Network.

RGV is led by the Plant Genetic Resources Curatorship system, which coordinates germplasm banks and plant collections at a strategic level, allowing the planning of midterm (5 years) conservation actions. RGV consists of 20 component projects, of which 11 are dedicated to the conservation activities of genebanks and the other nine are of transversal actions (Fig. 1). RGV is linked to several other Embrapa's portfolios and project arrangements, considering that conserved genetic resources form the basis for several research projects.^9^

Embrapa's RGV is fed by projects with specific activities for enrichment, conservation, characterization, and documentation of the collections, with the purpose of making them available for research, improvement, and food safety. Currently, Embrapa's plant genetic resources are conserved in (1) active genebanks (AGs), (2) long-term seed bank (Colbase), (3) *in vitro* bank, and (4) DNA bank.^10^ Colbase works as a safety duplicate of the accessions maintained in AGs, to conserve seeds at −20°C for a long period.^11^ Embrapa also develops research and innovation to subsidize and promote *in situ* and *on-farm* conservation, both in natural environments and in agroecosystems.^12^

### *Ex situ* conservation

*Ex situ* conservation of plant genetic resources refers to the maintenance of germplasm outside their place of origin in the short, medium, or long term. It comprises activities of enrichment through collection and exchange, documentation and proper conservation. *Ex situ* plant conservation at Embrapa works on germplasm acquisition through collection expeditions, exchange with institutions in Brazil and abroad, documentation, and development/improvement of methods related to *ex situ* conservation. The main aim is to do research on the establishment and improvement of methodologies used for *ex situ* collection and conservation of plant genetic resources, to ensure the physical, physiological, and genetic integrity of the collections, and also to provide the information associated with them.^13^

**FIG. 2.**
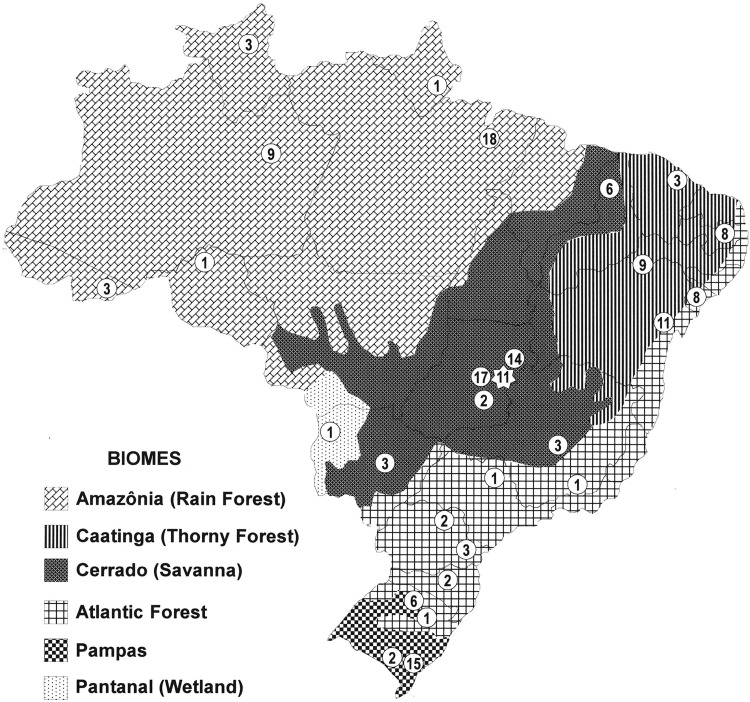
Distribution of AGs by Brazilian States and Biomes. The *values within circles* are the numbers of AGs located in 29 of Embrapa's decentralized units. The *star* in the center of the map represents Embrapa Genetic Resources and Biotechnology (CENARGEN) where the Seed Base Collection (Colbase) and *in vitro* and DNA banks are preserved at BGen. AGs, active genebanks; BGen, Genetic Bank of Embrapa. Data from Azevedo and Bustamante.^10^

The AGs of Embrapa's RGV are organized in groups of (1) Cereals and Pseudocereals; (2) Oilseeds, Fibrous, and Leguminous; (3) Vegetables and Condiments; (4) Forages; (5) Fruits; (6) Medicinal, Aromatic, Dyes, and Insecticides; (7) Ornamental; (8) Forestry and Palm Trees; (9) Industrial; and (10) Roots and Tubers.^10^ The most recent published global survey carried out by Embrapa reports ∼150,000 accessions of 470 genera and 1130 species (native and exotic) conserved in 134 AGs; 123,000 accessions of 620 genera and 735 species in the Colbase; 1250 accessions of 24 genera and 63 species in the *in vitro* bank; and 12,000 accessions of 16 genera and 21 species in the DNA bank^10^ (Table 1), involving ∼300 researchers from the different Embrapa units and ∼100 partner institutions. These germplasm collections are strategically distributed in different Brazilian regions and biomes (Fig. 2), in 29 Embrapa's centers, including *Embrapa Genetic Resources and Biotechnology*, where Colbase is being preserved in the new facility of the Genetic Bank of Embrapa (BGen), in Brasília-DF. It is estimated that at least 240 genebanks are maintained in the other SNPA institutions,^14^ such as Agência Goiana de Assistência Técnica, Extensão Rural, e Pesquisa Agropecuária (EMATER); Empresa de Pesquisa Agropecuária de Minas Gerais (EPAMIG); Empresa de Pesquisa Agropecuária do Estado do Rio de Janeiro (PESAGRO); Empresa de Pesquisa Agropecuária e Extensão Rural de Santa Catarina (EPAGRI); Empresa Mato-grossense de Pesquisa, Assistência e Extensão Rural (EMPAER); Fundação Estadual de Pesquisa Agropecuária (FEPAGRO); Instituto Agronômico de Campinas (IAC); Instituto Agronômico de Pernambuco (IPA); Instituto Agronômico do Paraná (IAPAR); Instituto Nacional de Pesquisa da Amazônia (INPA); Universidade de Campinas (UNICAMP); and Universidade Federal Rural do Rio de Janeiro (UFRRJ).

**Table 1.** Numbers of Active Genebanks, Genera, Species, and Accessions Conserved in the Embrapa's Plant Genetic Resources Network

	*No. of*					
*Genebank group*	*Genebanks*	*Genera*	*Species*	*Accessions*	*Institutions*	No. of banks under FAO treaty^*^
Cereals and Pseudocereals	12	16	88	59,408	5	10
Oilseeds, Fibrous, and Leguminous	21	21	169	48,323	9	10
Vegetables and Condiments	18	42	72	10,878	3	1
Forages	14	108	195	9284	11	2
Fruits	46	74	236	8693	14	5
Medicinal, Aromatic, Dyes, and Insecticides	15	86	103	4108	7	0
Ornamental	5	90	153	512	4	0
Forestry and Palm Trees	12	24	56	3359	6	0
Industrial	8	8	19	2631	5	0
Roots and Tubers	10	3	38	5232	7	9
All active genebanks	161	472	1129	15,2428	29	37
Long-term seed bank (Colbase)	1	623	735	12,3261	1	^*^
In Vitro Bank	1	24	63	1258	1	^*^
DNA Bank	1	16	21	12,000	1	^*^

Data from Azevedo and Bustamante.^10^

^*^ Species listed in Annex 1 of the FAO treaty, which are covered by the Multilateral System. Since Brazil is a signatory to the treaty, the accessions of these species must be available via Standard Material Transfer Agreement (SMTA).

The DUs that house the largest number of AGs are, in general, ecoregional centers that operate in regions that embrace large biomes such as *Embrapa Eastern Amazon* (with 18 AGs) in the State of Pará (North Region), *Embrapa Temperate Agriculture* (15) in the State of Rio Grande do Sul (Southern Region), and *Embrapa Cerrados* (14) in the State of Goiás (Central-West Region). On the contrary, some Commodities Centers, which work with a large number of crops, are also responsible for maintaining many AGs, such as *Embrapa Vegetables* (17 AGs) in Brasília-DF (Central-West Region) and *Embrapa Cassava & Fruits* (11) in Bahia (Northeast Region) (Fig. 2).

The biggest collections are rice (27,050 accessions), soybean (18,024), beans (16,447), wheat (15,118), and sorghum (7215). Some other collections are very small, such as some native fruits, coconut (36 accessions), and strawberry (20 accessions). It is important to highlight that those collections are maintained in the field or in greenhouses, and the focus is to conserve the genetic diversity of each accession. So, in some cases, there are >150 plants of each accession in the field, as in the case of coconut.

### Embrapa's Genetic Bank

**FIG. 3.**
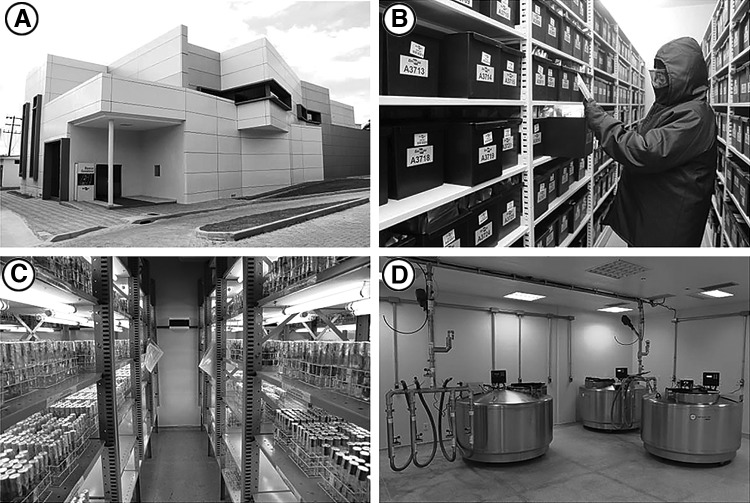
Infrastructure of Embrapa's Genetic Bank, inaugurated in 2014 at CENARGEN, Brasília-DF. **(A)** building; **(B)** seed cold chamber; **(C)** *in vitro* conservation room; and **(D)** cryopreservation facilities. Photographs **A** and **B**: Melo^15,17^; Photographs **C** and **D**: Brazil Agency - EBC.^18^

BGen, located at CENARGEN, in Brasília-DF, and inaugurated in April 2014, is a two-level building with a total area of >2000 m^2^, with modern and safe facilities to preserve, in the medium- and long-term, basic germplasm collections of plant and animal and backup of microorganism collections from Embrapa and partner institutions^15^ (Fig. 3). Plant germplasm is conserved in the form of seeds in cold chambers at −20°C, or in the form of vegetative structures by tissue culture or cryopreservation. BGen has laboratories, chambers for seed and *in vitro* plants conservation, cryopreservation tanks, and ultra-low temperature freezers for DNA banks. The facility for seed conservation is the largest in Latin America with the capacity to store 750,000 seed samples.^15^

The long-term seed storage capacity of the genebank ensures that all Embrapa's AGs can be duplicated in this structure. *In vitro* conservation and cryopreservation are performed on demand and for species for which protocols have already been developed. Researches to develop new protocols are always in progress. Also, species for which seed conservation is not feasible are prioritized. DNA conservation is also done on demand. This demand may come from ongoing research projects at Embrapa that study population genetics of genetic resource species as well as, and especially, from Colbase seed storage. All material entering the Colbase seed storage has the germination test performed. At least 10 plants of each accession are sent for DNA conservation.

One of the objectives of BGen is to house collections of plants, animals, and microorganisms maintained by partner institutions in Brazil and in other countries, such as the backup of the *in vitro* potato collection of International Potato Center (CIP).^16^ From 2014 to 2016, the complete CIP's collection with 4391 accessions was safety duplicated from Peru to BGen, Brasília (back box security copy).^16^ It is the world's most valuable potato collection in terms of genetic diversity, containing both wild and cultivated varieties.

### *In situ* and *on-farm* conservation

*In situ* and *on-farm* conservation is essential in conserving genetic resources and complements *ex situ* conservation. It covers the conservation, management, and restoration of species populations and their associated ecosystems, ensuring the evolution of species over time, in response to changing environmental conditions and the maintenance of agrobiodiversity. Embrapa develops research and innovation to promote conservation, both in natural environments and in agroecosystems, conducting biological inventories and geographic analysis for conservation planning; evaluation and development of management techniques for the sustainable use of biodiversity; ecological restoration in degraded landscapes; analysis and promotion of conservation of genetic resources by local communities and farmers.^19^ These studies subsidize actions and public policies for conservation, aiming not only the maintenance of species and their habitats but also of the human communities and their ways of life.^12,19^

In the case of *in situ* conservation, important advances were made to create and establish conservation units (CUs) to protect natural areas that are threatened to disappear, such as large areas of forest in the Amazon biome. Furthermore, other important government initiatives, such as research conducted to indicate priority areas for *in situ* management and the prospecting of native species with potential for sustainable use, have arisen from the ratification by Brazil of the Convention on Biological Diversity in 1994.^4^ To achieve these objectives, the National System of Nature Conservation Units (SNUC) was established in 2000, coordinated by the Ministry of the Environment (MMA), which has promoted the creation and management of CUs at the municipal, state, and federal levels, allowing an overview of the natural areas to be preserved.^20^ In addition, SNUC has been establishing mechanisms that regulate the participation of the society in the management of CUs, enhancing the relationship between the State, citizens, and the environment.

**FIG. 4.**
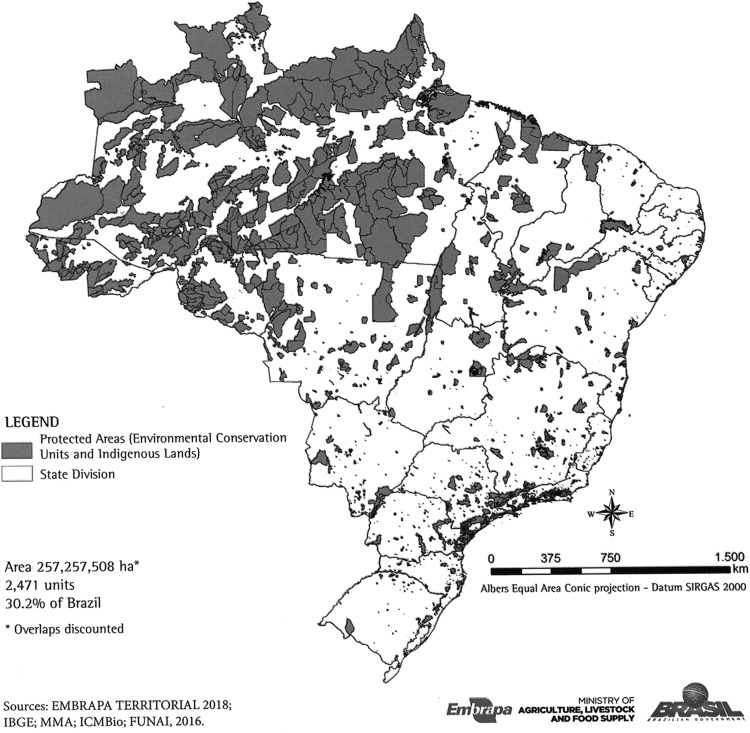
Distribution of legally protected areas in Brazil, including conservation units and indigenous lands. Source: Miranda.^22^

**FIG. 5.**
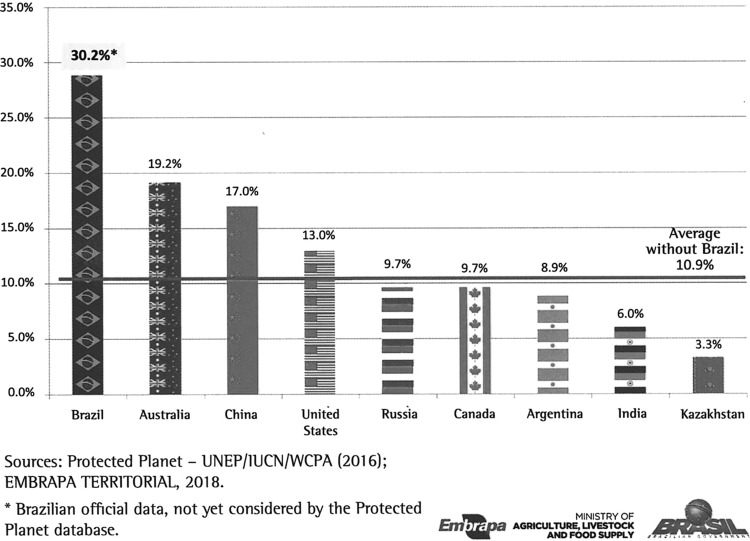
- Percentage of protected areas in countries with >2.5 million km.^2^ Data from Miranda.^22^

The SNUC is the set of CUs that differ in two broad groups according to the form of protection and permitted uses: (1) those that need greater care due to their fragility and particularities (CU of Full Protection), and (2) those that can be used sustainably (CU of Sustainable Use).^21^ Internationally, protected areas (PAs) are designated as lands for both CUs and traditional populations, such as indigenous lands (ILs).^22^ From the union of these two categories in Brazil, there are a total of 2471 units (1871 CUs + 600 ILs) covering an area equivalent to 30.2% of the Brazilian territory^22^ (Fig. 4). The relative distribution of PAs in the 26 Brazilian States shows that the percentage of PAs varies greatly from 71 at Amapá's State (North) to 0.9 at Paraíba (Northeast). Table 2 shows that Amazonia is the biome with the highest occupation of PAs (51%), whereas Pampas is the one with the lowest occupation (3%).^22^ According to the International Union for Conservation of Nature (IUCN), the nine countries with an area of >2.5 million km^2^ devote, on average, 10% of their territories to PAs; and Brazil is the world champion in environmental protection (Fig. 5). In addition, PAs in Brazil cover areas with great economic potential, which is not the case in most other countries, where they cover bad and desert areas.^22^

**Table 2.** Absolute and Relative Distribution of Protected Areas in the Six Main Brazilian Biomes

*Biome*	*Area (ha)*	*Protected areas (ha) (CUs + ILs)*	*% of protected areas*
Amazônia (Rain Forest)	418,245,755	212,115,240	50.7
Cerrado (Savanna)	203,938,059	24,961,235	12.2
Atlantic Forest	110,613,570	10,020,980	9.1
Caatinga (Thorny Forest)	82,652,523	6,499,874	7.9
Pantanal (Wetland)	15,131,294	762,120	5.0
Pampas	17,776,394	506,755	2.9
Brazil	848,357,596	254,866,204	30.0

Values calculated without overlapping. Data from Miranda.^22^

CUs, conservation units; ILs, indigenous lands.

To complement *in situ* conservation, the RGV has two component projects, one to promote the interface between *in situ* and *ex situ* conservation strategies, and the other to support collection expeditions to get cultivated species, wild species, and neglected species, in areas prone to any kind of risk or in areas considered GAPs in the genebanks, whose diversity is not yet represented in *ex situ* collections.^9^

### Germplasm exchange

The basic purpose of the plant germplasm exchange system is to promote the import, export, and internal transit of plant propagation material for agricultural research in a safe manner. The introduction of germplasm in Brazil is a dynamic process that aims to obtain more productive varieties resistant to pests and adapted to the soil and climatic conditions of the country. CENARGEN coordinates the exchange of plant germplasm in harmony and cooperation with the Ministry of Agriculture, Livestock and Food Supply (MAPA).^23^

CENARGEN has been exchanging and quarantining imported germplasm since 1976.^24^ By December 2014, a total of 660,139 accessions were exchanged, of which 482,713 were imports, 67,871 exports, and 97,558 domestic exchanges^25–27^ (Table 3). Therefore, ∼17,000 accessions per year were exchanged in this period, the great majority of which refers to imports. The most exchanged crops during this period were corn, wheat, rice, vegetables, soybeans, and beans. International institutions that have sent more germplasm to Brazil include the International Maize and Wheat Improvement Center (CIMMYT, Mexico), the International Center for Tropical Agriculture (CIAT, Colombia); the United States Department of Agriculture (USDA), the CIP (Peru), the International Rice Research Institute (IRRI, Philippines), and the Commonwealth Scientific and Industrial Research Organization (CSIRO, Australia). The countries that received the most germplasm from Brazil in descending order were United States, Haiti, Peru, France, and Colombia. In Brazil, the institutions that received the greatest quantities of germplasm from abroad were Embrapa (several centers), the Agronomic Institute of Paraná (IAPAR), the Agricultural Research and Rural Extension Company of Santa Catarina (EPAGRI), the Universidade Estadual Paulista (UNESP), and the School of Agriculture “Luis de Queiroz” (ESALQ).^4,24–27^

**Table 3.** Number of Accessions Exchanged by Embrapa/CENARGEN from 1976 to 2014

*Period*	*Import*	*Export*	*Internal transit*	*Total*
1976–1979	11,338	4706	8720	24,764
1980–1984	52,360	8786	16,297	77,443
1985–1989	53,598	15,558	16,967	86,123
1990–1994	61,844	12,912	9159	83,915
1995–1999	84,078	7949	10,448	102,475
2000–2004	90,831	2021	10,079	102,931
2005–2009	94,830	9271	9994	127,001
2010–2014	33,834	6668	15,894	55,487
Total	482,713	67,871	97,558	660,139

Sources: Marques and Marinho,^25^ Ferreira and Silva,^26^ and Visualization of component project.^27^

Brazil is a megadiverse country and, although it is one of the most biodiverse countries in the world, Brazilian agriculture is very dependent on exotic plant genetic resources. Therefore, the exchange and more properly the importation of germplasm have shown to be quite efficient in the enrichment of the genebanks and in the supply of raw material to the plant breeding programs. In this context, almost a half million accessions have been imported from other countries, supplying both the various centers of Embrapa and other scientific institutions and universities in Brazil.^26^

Although several species native to Brazil are used as human food, most of them are fruits, nuts, or tubers and roots. Brazil is mainly dependent on exotic germplasm for food. Nevertheless, native materials such as groundnut, cashew, cassava, and pineapple are of interest to other countries. However, the export of Brazil germplasm is very low. This can be explained by very restrictive Brazilian laws. The law number 13,123/2015, currently in force,^28^ defines genetic resources as the genetic information of plants, animals, and microbial species, or any other species, including substances originating from the metabolism of these living organisms. This strict regulation also does not allow the definitive transfer of native genetic resources to other countries or international centers. This makes clear the difficulties faced when negotiating the material exchange agreements. It is also important to highlight that exotic material can easily be exchanged if not considered adapted (when a species forms spontaneous populations and acquired distinctive characteristics in this population) or when considered a landrace. Furthermore, all research with native species must be registered in the National System for Genetic Heritage (SisGen)^29^ before any publication or before the development of any commercial product arising from the use of native genetic material. Decree number 8,772/2016, in what concerns this registration, requires that the origin of the genetic heritage found under *in situ* conditions be informed, even if obtained from *ex situ* or *in silico* sources. Therefore, the Law and Decree that regulate access and benefit sharing in Brazil have already recognized access to dematerialized genetic resources in its framework, although this discussion is still ongoing in international level. Thus, the regulation of Law 13,123 provides that to submit a sequence to any database, a Standard Material Transfer Agreement (SMTA) with the database must be signed. Also, research utilizing genetic information obtained *in silico* must register the use of this information at SisGen if the research results are in any publication or product.^30^

## Embrapa's Databases on Plant Genetic Resources

### Background

In the late 1980s, Embrapa/CENARGEN started the development of the Genetic Resources Information System (SIRG), which had a centralized database format with decentralized maintenance and access.^31^ Due to limitations to expand computer equipment, the system was restricted to CENARGEN, preventing its full implementation. In 1996, SIRG underwent a reengineering work, incorporating modern information technologies, used at the time, by USDA's Germplasm Resources Information Network (GRIN), resulting in the development of the Brazilian Genetic Resources Information System (SIBRARGEN), designed to establish and maintain a centralized database on genetic resources of plant, animals, and microorganisms, and making it available through the Internet for searching the following databases: Taxonomy, Passport, Exchange, Quarantine, *ex situ* Conservation, Collection, Characterization, Evaluation and Use of Germplasm, Curatorship and Germplasm Banks.^31^ The SIBRAGEN homepage was designed with web tools (HTML and JSP) using Embrapa's network infrastructure. In its first phase, the system was implemented within Embrapa. However, other Brazilian institutions, within the SNPA, also have used it.^7,31^

About 8 years using SIBRARGEN, Embrapa decided that it should be technically evaluated to adapt to future needs, both to increase user satisfaction and to improve system performance by incorporating up-to-date Web technologies. At that time, Brazil had been selected by Food and Agriculture Organization (FAO) of the United Nations to join the group of countries that would test the integration of national genetic resources information systems through a multilateral system under development by FAO.^7^ This was done to meet the demand for germplasm exchange based on the MultiCrop Passport Data (MCPD-FAO) in the framework of the International Treaty on Plant Genetic Resources for Food and Agriculture (FAO Treaty). In this pilot test, SIBRARGEN made adjustments to comply MCPD standards and made available passport data of the cassava genebanks. Another important aspect considered in the framework of the system update was the integration of SIBRAGEN Plant Module into Global GRIN (Germplasm Resources Information Network), developed by the USDA in cooperation with Biodiversity International and the Global Crop Diversity Trust.^7^

For at least three decades, Embrapa used the SIBRARGEN as the main database for all genetic resource activities, such as germplasm exchange, quarantine, and long-term conservation, carried out by Embrapa/CENARGEN. Over the years, CENARGEN has ceased to be the main responsible for the collection, conservation, regeneration, and germplasm distribution activities, which are being increasingly carried out by the curators of AGs in the Embrapa's DUs. By now, 29 DUs are involved in Plant Genetic Resources Conservation, from collection up to characterization. With this decentralization, it became necessary to develop a more modern, user-friendly, and accessible online tool that would allow the curators, in any of the DUs, to feed the system with the data related to the bank under their responsibility. CENARGEN, in this new scenario, remains responsible for coordinating the entire conservation system of Embrapa for quarantine, germplasm exchange, and for the long-term collection (Colbase).

### Alelo: Embrapa's genetic resources portal

In view of the need for a new, more modern, and accessible documentation system, Embrapa, in 2011, began to develop the Alelo System. This data platform encompasses all the information related to the conservation of plant, animal, and microbial genetic resources at Embrapa.^32^ The system is designed to document, record, and make data publicly available. Everything about passport data, routine activities and characterization and evaluation can be documented in this system.^33^ It was developed in free software and is available on the Internet (http://alelo.cenargen.embrapa.br). It allows quick and easy access to the general public. It also includes information on quarantine, germplasm exchange, DNA, long-term conservation, *in vitro* conservation, and the herbarium.^34^ Alelo is populated by the curators and their teams in a decentralized manner, allowing the updating of the Embrapa genetic resources database.

All the information about Embrapa's AGs is being migrated to Alelo platform. It is expected that all the passport data will be available for consultation by the end of 2018 and all information regarding characterization will be available by the end of 2019. Table 4 shows the number of genebanks and accessions that, to date, have already been migrated to the Alelo database. Comparing the data reported in this table with Table 1 (complete inventory), we can see that 70% of the AGs are already registered in the Alelo, but only 55% of the accessions were transferred. On the contrary, the upgrade and migration of Colbase are more advanced with 75% of the accessions transferred to the Alelo.

**Table 4.** Active Genebanks and Long-Term Seed Bank (Colbase) Registered in the Embrapa's AleloVegetal Database and Publicly Shared at Alelo's Portal on April 2018

	*No. of*		
*Institution*	*Genebanks*	*Accessions*	*Genebank names*
Carlos Gayer Research Center	1	25	Kiwi
Embrapa Tropical Agroindustry	3	930	Amaryllidaceae; Cactaceas; Cashew
Embrapa Cotton	6	1869	*Carthamus*; Castor bean; *Cnidosculus*; Peanuts; Sesame; Sisal
Embrapa Amapá	3	183	Buriti; Cupuassu; Inajá
Embrapa Western Amazon	6	1720	Cassava; *Croton*; Cupuassu; Guaraná; Native Fruits; Oil Palm
Embrapa Eastern Amazon	13	1183	Bacuri; Brazil Nuts; Camu-Camu; Cassava; Cupuassu; Curauá; *Heliconia*; *Hevea*; Ipeca; Jaborandi; Piperacea; Taperebá; Urucum
Embrapa Rice & Beans	2	32,790	Beans; Rice
Embrapa Cerrados	3	285	Cassava; Macaúba; *Passiflora*
Embrapa Temperate Agriculture	13	2696	Azevem; *Capsicum*; Carrot; Cucurbitaceae; Espinheira-Santa; Leguminous *Forages*; Native Fruits; Onion; Ornamentals; Potato and Wilds; Prunoids; Strawberry; Sweetpotato
Embrapa Forest	1	578	Forest species
Embrapa Beef Cattle	2	630	*Panicum*; *Stylosanthes*
Embrapa Vegetables	12	8721	Sweet potato; *Capsicum*; Cucurbitaceae; Eggplant; Garlic; Lettuce; Melon; Onion; Pepino; Tomate; Vegetables; Wild Solanum
Embrapa Cassava & Fruits	7	3659	Acerola; Banana; Bromelias; Cassava; *Manihot*; Papaya; Pineapple
Embrapa Mid-North	4	472	Babassu; Cajuí; Cowpea; Mango
Embrapa Maize & Sorghum	3	4513	Maize; Millet; *Sorghum*
Embrapa Pantanal	1	55	Native Forages
Embrapa Southeast Livestock	1	392	*Paspalum*
Embrapa Southern Livestock	2	542	Leguminous Forages; South Forages
Embrapa Genetic Resources and Biotechnology	3	828	*Arachis*; Cultivars; Fava
Embrapa Roraima	1	66	Orchids
Embrapa Semiarid	7	692	Amburana; *Cenchrus*; Grape; *Macroptilium*; *Manihot*; Onion; *Spondias*
Embrapa Soybean	2	265	Pupunha; Sunflower
Embrapa Coastal Tablelands	4	581	Coconut; *Desmanthus*; Jenipapo; Sugarcane
Embrapa Wheat	6	17,403	Barley; Canola; Oat; Rey; Triticale; Wheat
Amazon Research National Institute	4	769	Camu-Camu; Cupuassu; Pupunha; Vegetables
State University of “Norte Fluminense”	1	408	*Capsicum*
All active genebanks	111	82,255	
Long-term seed bank (Colbase)	1	92,895	222 Seed banks (grouped by species or genus)

Source: http://alelo.cenargen.embrapa.br/

In recent years, CENARGEN, along with Ministry of Agriculture, Livestock and Food Supply (MAPA), started to share the use of the Alelo platform with other Brazilian research institutes and universities. This can be considered the first step toward organizing and making available all the information regarding the public collections in Brazil. CENARGEN has already signed a membership and responsibility agreement with Rio de Janeiro State Agricultural Research Corporation (Pesagro), Rural Federal University of Rio de Janeiro (UFFRJ), State University of North Fluminense (UENF), State Agricultural Research Foundation (FEPAGRO-RS), and National Institute of Research of the Amazon (INPA). With the agreement, the parties are committed to exchanging confidential and nonconfidential information uploaded, downloaded, and shared through the Alelo portal.^34^

These partnerships are not restricted to national institutions. Teams of curators from Uruguay's National Agricultural Research Institute (INIA) and the Paraguayan Institute of Agrarian Technology (IPTA) have also signed the same agreement, which allows them to perform a range of activities and duties. To work with Embrapa's teams, the curators in partner organizations participate in training on Alelo's reference modules: passport, observation, conservation, and studies for system data validation.^34^

### Compatibility of Alelo with global databases

A cooperation agreement signed between Embrapa and the Global Crop Diversity Trust will enable the automatic migration of public data on plant genetic resources generated by Embrapa to the information system Genesys (https://www.genesys-pgr.org), a global information portal on genetic resources, which currently houses genebanks data from ∼200 countries, covering ∼11 million records, including passport, collection, characterization, and evaluation. Since Genesys data can be migrated to GRIN Global, the Alelo is compatible with GRIN Global. The main objective is to comply with the requirements of the International Treaty on Plant Genetic Resources for Food and Agriculture (ITPGRFA), ratified by Brazil in 2006.^33^

Embrapa's IT team has developed a tool that allows the automatic migration of information from the Alelo to the global portal. The data to be shared are those updated and validated by the curators of the Actice Genebanks and/or work collections and already made available to the public. In addition to facilitating compliance with ITPGRFA, automatic migration will facilitate the work of the teams involved, which often have to report the same data to different institutions, generating rework. The first stage of this automatic migration will take place in April 2018, and will be held for Genesys and WIEWS-FAO databases (Personal communication, Gilberto Hiragi, 2018).

### Herbarium of Embrapa

Embrapa's Herbarium, located at CENARGEN, was established in 1976, and is registered in the Index Herbariorum (http://sweetgum.nybg.org/science/ih/) with the code CEN. In March 2005, it was accredited as a Faithful Custodian by the Genetic Patrimony Management Council of the Brazilian Ministry of Environment (CGEN/MMA). Since 1985, it has been made available online. (http://plantwall.cenargen.embrapa.br/elcen2web/elc2html/elc2banco01.asp), and in 2014 its collection is available in the Virtual Herbarium of Flora and Fungi-INCT (http://inct.florabrasil.net/).

CEN Herbarium and its Plant Systematics Laboratory aim to study and record species of Brazilian flora and local flora, carry out expeditions to collect germplasm and support *ex situ* and *in situ* conservation actions. Currently, it has ∼108,000 exsiccates and is one of the most representative in Brazil for the Cerrado biome. It also houses growing collections of native plants and wild relatives of Caatinga and Amazon, as well as species of economic interest, including forage grasses and legumes, cassava, peanuts, pineapples, yams, oleaginous, ornamental, medicinal, forest and wild relatives of cultivated plants. Since 2017, data and images from this herbarium are available in the Global Biodiversity Information Facility (GBIF) (https://www.gbif.org), which is the largest online database on global biodiversity.^35^

## Final Considerations

Over the last 40 years, Embrapa has developed a large and complex system for the conservation of plant genetic resources. As a National Agricultural Research Institution, it took over the responsibility of conserving >140 different crops and wild relatives, allowing Embrapa to access a very rich source of resources that culminated in the development of hundreds of projects and consequently hundreds of cultivars, patents, and processes. They all had and still have conserved genetic resources as the basis of the research.
